# Role of geriatric intervention in the treatment of older patients with cancer: rationale and design of a phase III multicenter trial

**DOI:** 10.1186/s12885-016-2927-4

**Published:** 2016-12-01

**Authors:** Pierre Soubeyran, Catherine Terret, Carine Bellera, Franck Bonnetain, Olivier Saint Jean, Angéline Galvin, Camille Chakiba, Marie-Dominique Zwolakowski, Simone Mathoulin-Pélissier, Muriel Rainfray

**Affiliations:** 1 Department of Medical Oncology, Institut Bergonié, Comprehensive Cancer Center, F-33000 Bordeaux, France; 2 University of Bordeaux, F-33000 Bordeaux, France; 3 French National Cancer Institute (INCa) Integrated Cancer Research Site (SIRIC), Institut Bergonié, Comprehensive Cancer Center, F-33000 Bordeaux, France; 4 Department of Medical Oncology, Centre Léon Bérard, Regional Comprehensive Cancer Center, Claude-Bernard Lyon-1 University, Lyon, France; 5 Clinical and Epidemiological Research Unit, Institut Bergonié, Comprehensive Cancer Center, F-33000 Bordeaux, France; 6 Inserm, CIC-EC14.01 (Clinical Investigation Centre – Clinical Epidemiology Unit), F-33000 Bordeaux, France; 7 Inserm U1219 Research Centre, Epicene Team (Epidemiology of Cancer and Environmental Exposure) University of Bordeaux, Bordeaux, 33076 France; 8 Methodology and Quality of life in Oncology Unit, EA3181. CHU Besançon, F-25000 Besançon, France; 9 Geriatric service, Georges Pompidou European Hospital, Paris, France; 10 Department of Gerontology, Centre Hospitalier Universitaire, Bordeaux, France; 11 Medical Oncology, Institut Bergonié, Comprehensive Cancer Center, 229 cours de l’Argonne, 33076 Bordeaux, France

**Keywords:** Aged, Elderly, Cancer, Geriatric intervention, Case-management, Chemotherapy, Clinical trial

## Abstract

**Background:**

In the general geriatric population, programs linking geriatric evaluation with interventions are effective for improving functional status and survival of the patients. Whether or not these interventions improve health related quality of life (HRQoL) or overall survival (OS) in older patients with cancer is not yet clear. Indeed, randomized data on the effect of such interventions on survival and HRQoL are rare and conflicting. We describe the rationale and design of a phase III multicenter trial aimed at assessing the efficacy of geriatric intervention in the management of elderly patients with cancer.

**Methods/design:**

Approximately 1200 patients, 70 years and older, considered in need of a geriatric intervention based on the G8 screening tool will be randomized into two intervention arms. The ‘Usual-care’ arm involves standard oncological care based on pre-defined oncological protocols. In addition to the standard oncological care, the ‘Case-management’ arm involves a multidimensional geriatric assessment and interventions tailored for the patient. Efficacy will be assessed using a co-primary endpoint encompassing OS and HRQoL.

**Discussion:**

This trial has been designed to assess whether focused geriatric case management can either improve OS or HRQoL in elderly cancer patients considered in need of geriatric assessment.

**Trial registration:**

Clinicaltrials.gov ID: NCT02704832.

## Background

As the worldwide population ages and progress in cancer treatment increases survival, improving the management of older patients with cancer has become a major public health issue. Significant progress has been made in recent years, and geriatric assessment tools with prognostic value to identify specific problems in the elderly cancer population have been developed and validated [1–4]. However, this approach takes time and skills to administer and interpret, and only a few teams in France can perform full geriatric assessment in routine practice for all elderly cancer patients. We have developed [5] and validated the G8 screening tool [3], a short, easy-to-administer screening tool to identify patients in need of comprehensive geriatric assessment (CGA) and specialized interventions. However, the impact of further geriatric interventions on elderly cancer patient outcomes has not yet been investigated.

A number of randomized trials on geriatric interventions have shown benefit in various aspects for older cancer patients such as physical activity levels or health related quality of life (HRQoL) (Table 1). Goodwin et al. reported that nurse case management had a positive impact on the management of elderly patients with breast cancer [6]. Morey et al., in a 12-month home-based study, reported the impact of physical activity in overweight, long-term cancer survivors older than 65 years of age. They reported gains in function scores, basic lower extremity function, dietary habits and an improvement in the overall HRQoL [7]. In another study, an improvement in lean mass, muscle strength, walk time and HRQoL was observed with a resistance and aerobic exercise program for 57 prostate cancer patients with a mean age of 70 years [8]. Gains in practice of two or more goal behaviors, exercise minutes per week, total fat, saturated fat, and body mass index (BMI) were observed for 443 patients with newly diagnosed loco-regional breast or prostate cancer after a 10-month program of tailored mailed print materials [9]. Geriatric intervention reduced the pain in frail elderly patients who underwent elective surgery for a solid tumor but did not have any significant effect on delirium, complications, length of stay, care dependency and HRQoL [10].

**Table 1:** Literature review of geriatric intervention programs

First author	Study design	N	Population	Mean age	Intervention	Primary end-point	Results
Bourdel-Marchasson [13]	Multicenter RCT*	336	Patients with solid tumor treated by chemotherapy at risk of malnutrition (17 ≤ MNA ≤ 23.5).	78.0y	3–6 months diet counselling intervention	1-year mortality	- Early dietary counselling was efficient in increasing intake but had no beneficial effect on mortality.
Hempenius [10]	Multicenter RCT	260	Frail elderly patients undergoing elective surgery for a solid tumor	≈77.5y	Geriatric liaison intervention	Postoperative delirium	- Intervention for frail elderly cancer patients receiving surgery to prevent post-operative delirium was not effective.
Demark-Wahnefried [34]	Multicenter international RCT (RENEW study)	641	Overweight long-term survivors (≥5 years) of colorectal, breast and prostate cancer	≈73y	12-month diet and exercise intervention via telephone counseling and print materials	Change in functional status (baseline/12-month and 24-month) Diet quality, BMI and physical activity	- Significant change in functional status between intervention group and control group (*p* < 0.01): amelioration of functional decline in intervention group. Significant change in diet quality, physical activity and BMI (*p* < 0.01).
Morey [7]						Change in functional status (baseline/12 m) using the Medical Outcomes Study SF36 questionnaire, health-related QoL	- Significant change in physical function (*p* = 0.03) and QoL (*p* = 0.007) between groups.
Lapid [35]	Subset geriatric analysis from stratified, two-group RCT	33	New advanced cancer diagnosis (5-year OS: 0–50 %) planned to receive radiotherapy	≈72y	4-week multidisciplinary QoL intervention	QoL measured with Spitzer uniscale and linear analogue self-assessment (LASA) at baseline and weeks 4, 8, and 27	- Significant improvement in QoL (*p* < 0.05) at baseline, maintained at 4 and 8 weeks.
Rao [14]	Subset analysis from RCT [36]	99	Frail elderly cancer patients hospitalized on a medical or surgical ward (≥2 days)	≈74y	Geriatric assessment and patient management by a geriatric attending physician and a social worker	12-month survival and health-related QoL (after randomization), ADL, physical performance, health service utilization, and costs	- No significant effect on survival or QoL parameters. Positive effects of geriatric inpatient care on mental health and bodily pain (*p* < 0.05). Days of hospitalization and cost similar.
Goodwin [6]	Multicenter RCT	335	Older women (≥65y) newly (<2 months) diagnosed with breast cancer	≈72y	12-month nurse case management	Type and use of cancer-specific therapies received in the first 6 months after diagnosis. Patient satisfaction and arm function	- More appropriate management for women receiving nurse case management (Breast-conserving surgery, adjuvant radiation, radiation therapy, axillary dissection and breast reconstruction surgery). Better arm function and higher satisfaction in intervention group.
McCorkle [15]	Single centerRCT	375	Old patients (≥60y) newly diagnosed with solid cancer	60–92	4-week home-based case management by nurse	Length of survival	- Longer survival in intervention group than in usual care group (*p* = 0.001). Survival advantage for intervention group in late stage patients.
Galvao [8]	Two-arm single center RCT	57	Prostate cancer patient without bone metastases treated by AST (≥2 months)	≈70y	12-week progressive resistance and aerobic training (2/week) by an exercise physiologist	Muscle mass, strength, physical function, QoL	- Significant change in total body lean mass, muscle strength and endurance (*p* < 0.05). Change in QoL for general health (*p* = 0.022), vitality (*p* = 0.019) and physical health composite score (*p* = 0.02).

*QoL quality of Life, *RCT* randomized controlled trial

Geriatric intervention has also been shown to increase overall survival (OS) in the general elderly population in randomized controlled trials (RCTs) [11, 12]. However, only few RCTs have investigated the impact specifically for elderly cancer patients, with conflicting results. Bourdel-Marchasson et al. [13] found no impact on 1-year mortality after a nutritional intervention, and in a sub-analysis of a larger trial, Rao et al. [14] found no difference in 1-year survival after geriatric case-management for elderly cancer patients. An improvement in survival was reported in an RCT involving case-management in the form of a one-month nurse intervention for post-surgical elderly cancer patients, including 375 patients older than 60 years, but this benefit was restricted to the sub-population of patients with advanced disease [15].

Overall, results from various studies suggest that geriatric interventions can potentially improve OS in elderly cancer patients. Although several attempts are being made to identify more patients at risk that require geriatric interventions, any OS or HRQoL benefits following these interventions has not yet been sufficiently demonstrated.

Our objective is to conduct a two-arm phase III trial to assess the efficacy of geriatric intervention in the management of elderly patients with cancer (targeted oncology domains: breast, colorectal, lung, prostate, bladder, ovary, lymphoma). The trial will be restricted to patients with an abnormal G8 screening tool score that are considered in need of a geriatric intervention. This trial protocol was developed in collaboration with two national research platforms (Cancer and Elderly Platform and Cancer and Quality of Life Platform). We present here the key elements of the trial: objectives, study design, target population, description of the interventions, evaluation criteria and statistical methods.

## Methods/Design

### Objectives

#### Primary objective

The primary objective of the trial is to compare the efficacy of two treatment strategies “usual care” and “Case-management intervention” in elderly cancer patients considered in need of a geriatric assessment (G8 score ≤ 14). Treatment strategies are defined as follows:

- “Usual care”: Patients are treated according to ongoing standards in oncology.
- “Case-management intervention”: Patients are treated according to ongoing standards in oncology as in the “Usual care” arm. In addition, patients receive geriatric assessments and interventions coordinated by a geriatrician and a trained nurse, and tailored for the patient.

The intervention content for each arm has been precisely defined by a panel of experts in geriatric oncology. Efficacy will be assessed using a co-primary endpoint encompassing OS and three targeted HRQoL dimensions (global health status, physical functioning and emotional functioning as measured using the European Organization for Research and Treatment of Cancer, EORTC, QLQ-C 30 questionnaire [16]). Specifically, efficacy of the “Case- management intervention” will be considered superior to that of “Usual care” if, at one year, compared to usual care, improvement is observed for OS or at least one of the targeted HRQoL dimensions. We provide additional details of the primary endpoint in evaluation criteria and sample size calculation sections.

#### Secondary objectives

Secondary objectives are three-fold. First, in the population of elderly cancer patients considered in need of a geriatric assessment (G8 score ≤ 14), we will compare the two treatment strategies in terms of additional endpoints: 6-month objective response, 1- and 3-year progression-free-survival (PFS) and OS, HRQoL dimensions other than those targeted for the primary endpoint, grade 3–4 toxicities, duration and number of hospitalizations, place of residence (home versus nursing home or hospital).

Second, we will focus on the population recruited in the intervention arm. Our objective is to provide a description of geriatric parameters (evolution over one year, as well as descriptive data at baseline, 6 and 12 months): functional status, nutritional status, depression status, physical capacities, number and type of prescribed geriatric interventions.

Third, while our main focus is on elderly cancer patients considered in need of a geriatric assessment (G8 score ≤ 14), we also intend to provide a brief description of patients with a normal G8-score. Vital status, place of residence and disease progression at one year will be described.

### Overall study design

The efficacy of the treatment strategies “Usual care” and “Case-management intervention” will be assessed using a two-arm randomized multicenter trial, conducted in elderly cancer patients considered in need of a geriatric assessment (G8 score ≤ 14).

As additional objectives involve describing elderly cancer patients with a normal G8-score, a specific cohort will be built in parallel to the randomized trial. Patients initially screened for inclusion in the randomized trial but who will present with a G8 score ≤ 14 will be included in this parallel prospective cohort.

**Fig. 1:**
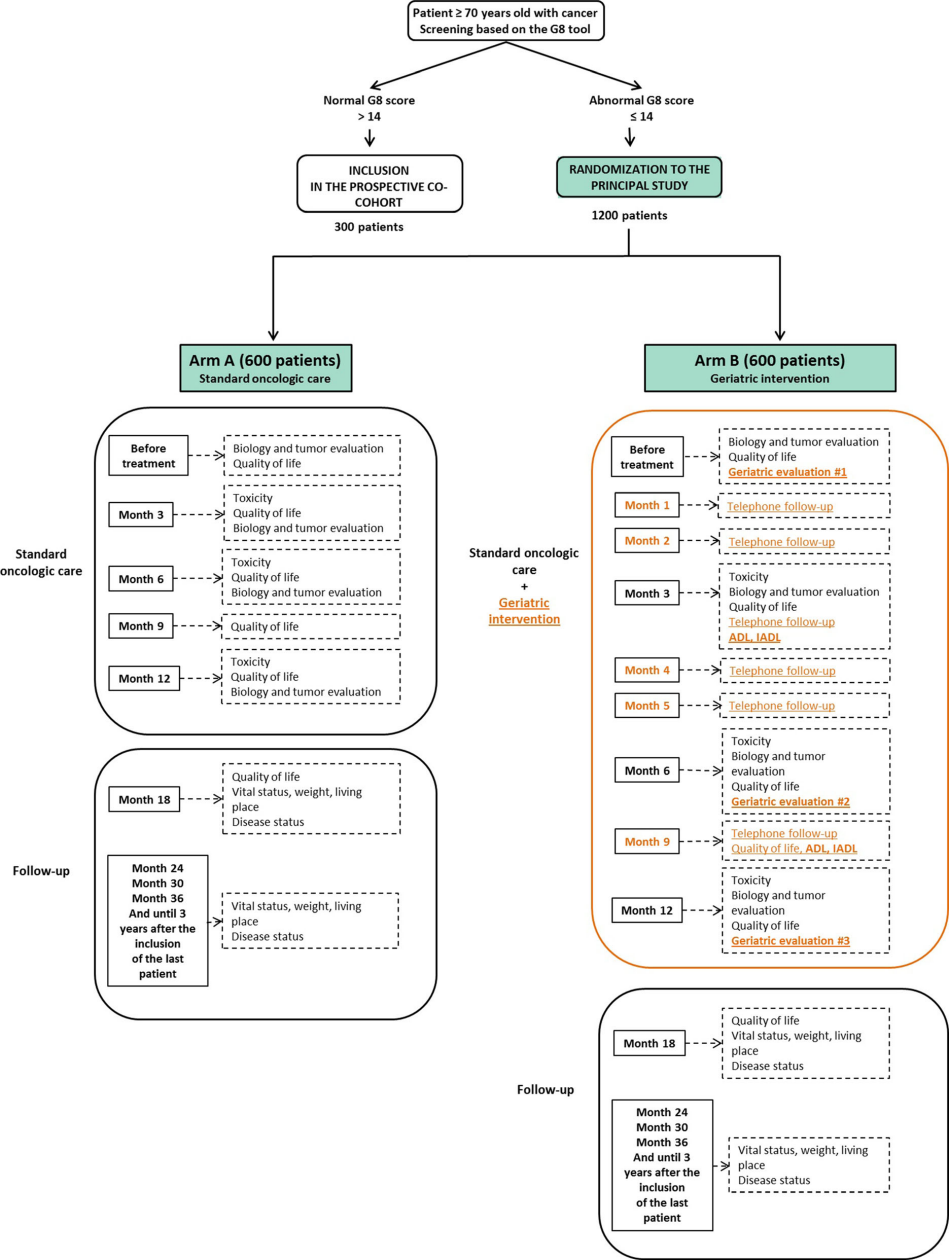
ᅟ

All elderly cancer patients treated at participating centers will first be screened with the G8 instrument (Appendix A). If the G8 score is altered (G8 score ≤ 14) then patients will be included in the main trial and randomized between “Usual care” and “Case-management intervention” (Fig. 1). For each arm, patients will be treated according to protocols specifically defined for this trial (See section “Description of the interventions”). If the resulting score is normal (G8 > 14) then patients will be included in the parallel cohort and treated according to the institution’s standard management process.

This study will be initially proposed to the 28 Regional Coordination Units for Geriatric Oncology accredited to the French National Cancer Institute across France, including Comprehensive Cancer Centers, teaching hospitals, public hospitals and private practices.

Data will be collected on an electronic case report form and directly input via the Internet. Data will be handled by online trial management software on the Internet; it will be transferred and monitored remotely in real-time. Similarly, randomization will proceed through internet-based software allowing rapid randomization at any time.

### Eligibility criteria

This study will include patients 70 years and older. In addition, patients must meet the following criteria: performance status 0 to 3 (WHO), candidate to 1^st^ line medical treatment (excluding best supportive care) of breast, colon, rectal, lung, prostate, bladder, or ovarian cancer and lymphomas (indolent and aggressive); excluding exclusive hormonotherapy (except prostate cancer); or, second line medical treatment (excluding Best Supportive Care) of breast, colon, rectal, prostate cancer, ovary and indolent lymphoma.

Exclusion criteria are life expectancy under 6 months, presence of any psychological, familial, sociological or geographical condition potentially hampering compliance with the study protocol and follow-up schedule, participation at the same time in another study in which investigational drugs are used, and patients who have already received two lines of treatment or for whom hormonal treatment (except for abiraterone acetate and enzalutamide in prostate cancer) or best supportive care is indicated.

### Description of the interventions

All patients included in the randomized trial will receive cancer care according to ongoing standards in oncology. The content of this intervention has been precisely defined in a *cancer care protocol* by a panel of experts in geriatric oncology, and depending on the disease and stage. For patients specifically randomized in the “Case-management intervention”, intervention will, in addition, involve multidimensional geriatric assessments and case-management geriatric interventions tailored for the patient. Similarly, this specific geriatric intervention has been precisely defined in a *geriatric care protocol* by a panel of experts in geriatric oncology. We first describe the development process for the *cancer care protocol* and the *geriatric care protocol* followed by details of the content of each protocol.

#### Development of the standard cancer care and geriatric intervention plans

The objective of this preliminary phase is to propose protocols for both *cancer care* and *geriatric care* for patients included in the randomized trial in order to ensure homogeneous oncological care (across treatment arms and between investigational sites) as well as homogeneous interventions in geriatrics (within the arm “Case-management intervention” and between investigational sites). Firstly, although the trial is aimed at assessing the efficacy of the geriatric intervention, it is important to acknowledge that for same disease, several cancer treatment options can be proposed, and dose adjustments are expected. By providing a *cancer care protocol*, clinicians will have a specific list of cancer treatment options for which they will be able to select an option. Secondly, it is important to ensure that patients who will be randomized in the geriatric intervention arm will be followed-up and treated according to a standardized procedures (use of the same questionnaires, same decision rules to propose specific interventions, etc.), justifying the development of a *geriatric care protocol*.

To develop and validate the final version of both the *cancer care* and the *geriatric care* protocols, two independent committees were set up for each protocol: a writing committee and a peer-review committee. For the *cancer care protocol*, the writing committee led by an oncologist (CT) consisted of seven additional experts in each targeted oncology domain (breast, colo-rectal, lung, prostate, bladder, lymphoma, or ovarian cancer), and the validation committee included four oncologists and one geriatrician. For the *geriatric care protocol*, the writing committee consisted of two geriatricians (MR and OSJ) and one nurse qualified in geriatric oncology (MDZ), and the validation committee included one oncologist and four geriatricians. The complete list of experts constituting the committees is provided in Appendix B.

Once the protocols were drafted by the writing committee, they were sent to the peer-review committee for assessment and comments with a standardized questionnaire consisting of seven questions on the clarity, appropriateness and applicability of the proposed protocols. Members provided a formal and advisory opinion on content and form of the initial version of the guidelines. Minor modifications to the protocols were incorporated following the review. Final documents were then redistributed to the writing committees for validation. This review process was an essential quality assurance step and the final document was circulated to all participating investigators of the trial and will serve as a reference document.

#### Description of the intervention for the arm ‘Usual care’

Patients will be treated according to the *cancer care protocol*, which provides, for each cancer site, a list of treatment options as well as guidelines for dose modifications and adverse event management. The complete *cancer care protocol* is available upon request.

Patients will be followed-up by the oncologist for three years after randomization. Baseline assessment includes clinical examination, laboratory tests (hematology, biochemistry and/or creatinine clearance), disease history and treatments, tumor assessment, and HRQoL assessment (QLQ-C30 and ELD-14). Quarterly visits by the oncologist during the first year will include a clinical examination, laboratory tests, tumor assessment, and HRQoL assessment (QLQ-C30 and ELD-14 questionnaires). Patients will then be seen every semester during the second and third years during which data on disease progression, HRQoL and vital status will be collected.

#### Description of the intervention for the arm “Case-management intervention”

Patients will be treated according to the oncological standards, as defined in the *cancer care protocol*. Patients’ follow-up with their oncologist will be identical to that of patients in the ‘Usual care’ arm. In addition, intervention will also involve case-management geriatric interventions tailored for the patient. The *geriatric care protocol* provides a full description of the case-management process which includes CGAs, telephone follow-ups by the nurse and patient file reviews by the geriatrician and the nurse.

The patient will be seen by the geriatrician and the nurse for CGA at baseline (after randomization and before treatment initiation), as well as 6 and 12 months after randomization. The CGA will include the following: questionnaires completed with the nurse (Activities of Daily Living (ADL) [17], Instrumental ADL (IADL), Mini Mental State Examination (MMSE) [18], Mini Nutritional Assessment (MNA) [19], Geriatric Depression Scale (GDS-15) [20], EORTC QLQ-C 30 [16] and the Elderly Cancer Patients version (EORTC QLQ- ELD14) [21]); social evaluation including mode of living, professional help, social situation; geriatric clinical consultation including Timed Up and Go (TUG) [22], Short Performance Physical battery (SPPB [23]), weight loss, subjective health, falls in last 12 months, co-morbidities, other medical treatment, pain (numerical scale) and sleep patterns; geriatric patient management plan defined by nurse and geriatrician. The geriatric management plan establishing any necessary interventions will be defined by the nurse and the geriatrician across eleven intervention areas according to the predefined rules in the *geriatric care protocol.* Table 2 presents a summary of these interventions for the eleven intervention areas: modification of therapy, balancing chronic diseases, pain treatment, nutritional intervention, physical activity, physiotherapy, psychological support, prevention of further cognitive impairment, treatment of sleeping disorders, assistance, and home help. For illustration, with regards to nutritional intervention, weight loss over the last three and six months should be assessed and the MNA questionnaire should be completed. The patient will be considered at risk of malnutrition in case of MNA score between 17 and 23.5 and in such case, advice on increasing dietary uptake (use of food pyramid adapted for elderly patients) should be provided as presented in the *geriatric protocol*. On the other hand, in case of an MNA score below 17, weight loss greater than 5% over the last three months or 10% over the last six months, then the patient will be considered malnourished, and should be referred to a dietician.

**Table 2:** Proposed geriatric interventions defined in protocol according to the eleven areas and screening tools used

Area	Indication	Intervention
Modification of therapy	Polypharmacy	• Re-evaluate treatment indications (with general practitioner), optimize treatment according to elderly patient protocols.
		• Use the STOPP-START* tool as a reference [37, 38].
Balancing chronic diseases	CIRS-G* Grade ≥ 3,	• Adaptation of therapy.
	arterial hypertension, diabetes, arthritis, or sensory problems	• Other non-drug interventions such as dietary advice, devices.
		• Referral to specialist doctor.
Pain treatment	Pain >1 on numeric scale	• Drug: WHO levels 1–3 antalgic treatment
		• Non-drug: physiotherapy, devices
Nutritional intervention	Malnutrition (MNA* ≤ 17)	• Referral to dietician (nutritional supplements, artificial nutrition, etc.)
	Weight loss ≥5 % over 3 m, or ≥10 % over 6 m	
	At risk of malnutrition - 17 < MNA ≤ 23.5	• Advice on increasing dietary uptake (use of food pyramid adapted for elderly patients)
Physical activity	Sedentary or at risk of falls	• Recommend daily walk, walking upstairs, carrying groceries
Physiotherapy	Difficulty with walking and balance (TUG*, and SPPB*). Fall in the last 12 m, weight loss, muscle loss	• Prescribe physiotherapy for muscular reinforcement Work on balance, and getting up from lying down position
		• Prescribe walking aids
Psychological support	GDS* ≥ 6	• Consultation with psychologist or psychiatrist
	Apparent anxiety (clinical assessment)	
	Antecedent of depression	
Prevention of further cognitive impairment	MMSE* < 24	• Behavioral monitoring during chemotherapy
		• Prevention of confusion [39]
Treatment for sleeping disorders	Positive screening score on adapted Epworth scale [40]	• Introduction, modification or discontinuation of treatment by hypnosis according to STOPP and START recommendations [37, 38]
Assistance	Social fragility identified (absence of social support)	• Ask patient directly if help is needed
		• Refer to a social worker
Home help	Difficulties performing daily tasks such as grooming, housework	• Prescribe support within the home (nursing, physiotherapy, housecleaner, etc.)

*STOPP-START Screening Tool of Older persons’ prescriptions, *CIRS-G* Cumulative Illness Rating Scale for Geriatrics, *MNA* Mini Nutritional Assessment, *TUG* Timed Up and Go, *SPPB* Short Performance Physical battery, *GDS* Geriatric Depression Scale, *MMSE* Mini mental state examination

Telephone follow-ups by the nurse will take place on a monthly basis for the first semester, then every three months up to one year, or after any event, such as hospitalization, or change of residence to nursing home. The following information should be recorded: clinical status of the patient including factors assessed at the 1-month review, any toxicity to treatment, social assistance in place, and coordination of future consultations.

Patient file reviews will take place after telephone follow-ups and will involve the geriatrician, the nurse and the oncologist in order to review how the geriatric interventions in place are progressing, and assess the need for further interventions according to the predefined rules (Table 2).

To standardize care in the “Case-Management arm”, a two-day training for nurses will be organized. Two nurses per investigating center will participate in the training which will be mandatory before the center can accrue. During this training session, nurses will be trained to perform the telephone follow-ups with patients.

#### Description of the intervention for patients included in the parallel cohort

Patients with a normal G8 score will be included in the parallel cohort and treated according to the institution’s standard management process.

### Evaluation criteria

Although OS is a usual endpoint to assess treatment efficacy in RCTs, it may not be sufficient to assess the treatment benefit in an elderly population. The effect on HRQoL should also be considered, since it may be as important as (and sometimes more than) duration of life for this population [24, 25]. Efficacy will be assessed using a co-primary endpoint encompassing OS and three targeted HRQoL dimensions including global health status, physical functioning and emotional functioning as measured using the EORTC QLQ-C 30 questionnaire [16]. Specifically, efficacy of the “case-management intervention” will be considered superior to that of “usual care” at one year, if there is a significant clinical improvement in OS without a significant deterioration in at least one of the targeted HRQoL dimensions, or if there is a significant clinical improvement in at least one of the targeted HRQoL dimension without a significant difference in OS.

OS is defined as the delay between the date of randomization and the date of death. If the absolute difference in OS at one year is at least 10% greater, then we will consider it as a significant improvement in the OS. For HRQoL, the three targeted dimensions will be assessed using the EORTC QLQ-C 30 questionnaire [16]. Dimensions will be scored according to the EORTC manual [26]. Each dimension will be scored ranging from 0 to 100 and a difference of 10 points or greater will be considered to be clinically significant [27]. If there is a major improvement in at least one of the three scales, without deterioration of the other two, then we will consider as a significant improvement in HRQoL. Secondary endpoints include PFS defined as the delay between the date of randomization and the date of progression or death whichever occurs first. Progression, as well as objective response, will be defined as per the RECIST criteria for solid tumors [28] or the Cheson criteria for lymphomas [29]. HRQoL dimensions other than those targeted for the primary efficacy endpoints will similarly be assessed based on the EORTC scoring manual, and the EORTC ELD-14 module will be assessed following published guidelines [30]. For each of the HRQoL dimensions, time until definitive deterioration (TUDD) will be investigated and defined based on a recent publication [31]. Toxicities will be graded according to the NCI-CTC v4.3 classification. For geriatric questionnaires (G8, ADL, IADL, MNA, MMSE, GDS-15, CIRS-G, TUG), cut-off scores used to define abnormal scores are those reported in the corresponding literature and summarized in Soubeyran et al. [3].

### Statistical methods

#### Randomization

For the phase III trial, randomization will be performed on an individual basis based on a 1:1 allocation ratio. Randomization will be stratified by investigational site, treatment line (first or second), and type of cancer (breast, colon, rectal or prostate cancer versus bladder, ovarian, or lung cancer or lymphoma). Because of the number of strata foreseen, randomization by minimization will be implemented [32].

#### Sample size

Sample size was estimated for the randomized trial. For the null hypothesis we assumed no difference in OS and no difference in the three targeted HRQoL dimensions between the two intervention arms. Alternative hypotheses were two-fold: improvement in OS or improvement in HRQoL. The 5% two-sided type-1 error rate was therefore split between the two alternative hypotheses with 2% for OS and 1% for each of the three HRQoL dimensions. Sample size improvement for testing improvement in OS assumed the following hypotheses: 2% (two-sided) type-1 error rate, 85% power, anticipated 1-year 50% OS rate in the ‘usual care’ group [13], 60% expected 1-year 60% OS rate in the “Case-management” arm (ie anticipated hazard ratio, HR = 0.74). These hypotheses led to an estimated sample size of 1096 subjects (548 per group; 486 events) needed to detect a difference in OS (log-rank test). Sample size for testing improvement in HRQoL was based on a 3% (two-sided) type-1 error. If the intervention is effective, we expect that it will allow us to detect, at one year, a mean difference of 10 points or more for at least one of the three targeted HRQoL scores (common standard deviation of 20 points). Each of the three scales will be independently tested. Five hundred and forty eight subjects per group (as needed for OS comparison) will provide sufficient power (>90%) for independently testing the three HRQoL scales at 1% (2-group t-tests of equal means – common variance).

To anticipate for non-assessable subjects (10%), 1200 patients need to be randomized (ie with G8 score ≤ 14). Based on previous research [5] about 80% of the patients are expected to have positive G8, thus 1500 patients need to be screened for inclusion in the randomized trial.

Since 20% are expected to have normal G8 score, about 300 patients will be included in the parallel cohort. Following our previous experience with the ONCODAGE validation study [3] which included 1668 patients over 18 months, we expect that 800 patients will be screened each year, leading to an expected inclusion period of 1.5 to 2 years. To reach all endpoints, three years of follow-up are required, giving total study duration of 5 years.

#### Statistical analyses

For the randomized trial, different analysis sets will be defined including the intent-to-treat (ITT) population (all randomized patients included and analyzed according to the allocated intervention), modified ITT population (mITT: all randomized patients with baseline HRQoL included and analyzed according to the allocated intervention) and per protocol population (PP: eligible patients and analyzed according to the intervention actually received). These three populations will be described according to the following characteristics: compliance with eligibility criteria, epidemiological characteristics, clinical and laboratory characteristics, treatment characteristics, and HRQoL. For the co-primary endpoints, the primary analysis will be conducted in the mITT population, with sensitivity analyses conducted in the PP population. OS will first be compared based on a log-rant test stratified on randomization factors (2% type 1 error rate). Additional analyses will be conducted including Cox regression modeling to provide an estimate of the effect size of the intervention, after checking for underlying model assumptions. For each targeted HRQoL dimension, a *t*-test assuming common variances will be conducted (1% type 1 error rate). Sensitivity analyses assuming a mean difference of 5 points or more for at least one of the targeted HRQoL scores will be conducted. In addition, variance analysis will be conducted for adjustment on randomization factors and additional covariates. Other endpoints will be analyzed using standard statistical techniques. PFS will be analyzed using the same methods as for OS. Censoring mechanisms for the analysis of TUDD will follow recommendations from Bonnetain et al. [28]. With regards to HRQoL data, missing data analysis will be performed to determine the missing data profile. Longitudinal models will be fitted to provide a description of the HRQoL over time and investigate effect of the intervention. For patients treated in the “Case-Management” arm, the analysis of geriatric parameters will involve summary statistics presented at various time-points as well longitudinal analysis to investigate trends over time.

### Monitoring and ethical considerations

The trial has been approved by the regional ethics committee (Comité de Protection des Personnes Sud-Ouest et Outre Mer III) and by the National Agency for Security of Medical and Health products (ANSM) and has been registered (Trial registration number: 2015-A01417-42). All patients will provide informed consent (both for the randomized trial and prospective cohort) and will be free to withdraw their consent at any time.

The study will be supervised and monitored continuously by a steering committee consisting of the principal investigator (oncologist), a coordinating geriatrician, a pharmacist in charge of the pharmacovigilance, a biostatistician, a data manager and the coordinating clinical research assistant. This committee will ensure the implementation and regular follow-up of the study, patient protection, ethical conduct of trial and evaluation of benefit/risk ratio. The scientific committee will include the steering committee as well as additional geriatrics, medical oncology, public health and biostatistics specialists. It will be responsible for validation modifications of the study protocol and reviewing scientific findings during the course of the study, providing patient protection, ethical conduct of trial and evaluation of benefit/risk ratio. An independent data monitoring committee (IDMC) has been foreseen and will be set up at least once during the course of the study.

## Discussion

Currently, the G8 is recommended for systematic use in France [33] to identify patients requiring increased clinical attention up to full geriatric assessment, and potentially a geriatric intervention plan. This generates a certain cost, for the CGA and for the geriatric interventions proposed, yet the gains in survival or HRQoL of these geriatric interventions have not yet been demonstrated. The current evidence to suggest that geriatric interventions have a positive impact on survival and HRQoL in elderly cancer patients is limited and conflicting, and randomized controlled trial data in this specific context are required.

The current study protocol proposes a two-arm randomized comparison trial of usual cancer care versus usual cancer care combined with geriatric intervention, in a group of elderly patients across multiple centers in France. Our randomized trial offers an innovative approach introducing a co-primary endpoint including both OS and HRQoL. This methodology integrates the HRQoL dimension in addition to life span, which may reflect elderly people expectations.

As such, we propose to compare efficacy using a co-primary endpoint encompassing OS and specific dimensions of HRQoL at one year – this is the first time this elderly-specific endpoint has been proposed in this context.

## Appendix

### Appendix A

**Table 3:** The G8 screening test

	Items	Possible answers (score)
A	Has food intake declined over the past 3 months due to loss of appetite, digestive problems, chewing or swallowing difficulties?	0 : severe decrease in food intake
		1 : moderate decrease in food intake
		2 : no decrease in food intake
B	Weight loss during the last 3 months	0 : weight loss > 3 kg
		1 : does not know
		2 : weight loss between 1 and 3 kgs
		3 : no weight loss
C	Mobility	0 : bed or chair bound
		1 : able to get out of bed/chair but does not go out
		2 : goes out
E	Neuropsychological problems	0 : severe dementia or depression
		1 : mild dementia or depression
		2 : no psychological problems
F	Body Mass Index (BMI (weight in kg)/(height in m^2^)	0 : BMI < 19
		1 : BMI = 19 to BMI < 21
		2 : BMI = 21 to BMI < 23
		3 : BMI = 23 and > 23
H	Takes more than 3 medications per day	0 : yes
		1 : no
P	In comparison with other people of the same age, how does the patient consider his/her health status?	0 : not as good
		0.5 : does not know
		1 : as good
		2 : better
	Age	0 : >85
		1 : 80–85
		2 : <80
	Total Score	0–17

### Appendix B: Writing and Validation Committees of the Cancer Care Protocol

**Writing Committee:** Catherine Terret (Medical Oncologist, Lyon, Chair), Thomas Aparicio (Gastro-enterologist, Paris), Etienne Brain (Medical Oncologist, Paris), Camille Chakiba (Medical Oncologist, Bordeaux), Stéphane Culine (Medical Oncologist, Paris), Gilles Freyer (Medical Oncologist, Lyon), Caroline Lalet (Central Clinical Research Assistant, Bordeaux), Hervé Le Caer (Pneumologist, Draguignan), Loic Mourey (Medical Oncologist, Toulouse), Pierre Soubeyran (Medical Oncologist, Bordeaux).

**Validation Committee:** Leila BENGRINE-LEFEVRE (Medical Oncologist, Dijon), Emmanuelle BOURBOULOUX (Medical Oncologist, Nantes), Nicole TUBIANA-MATHIEU (Medical Oncologist, Limoges), Simon VALERO (Geriatrician, Poitiers).

### Appendix C: Writing and Validation Committees of the Geriatric Care Protocol

**Writing Committee:** Muriel Rainfray (Geriatrician, Bordeaux, Co-Chair), Olivier St Jean (Geriatrician, Paris, Co-Chair), Camille Chakiba (Medical Oncologist, Bordeaux), Caroline Lalet (Central Clinical Research Assistant, Bordeaux), Marie-Dominique Zwolakowski (Advanced Practice Nurse, Bordeaux).

**Validation Committee:** Laurent Balardy (Geriatrician, Toulouse), Laurence CRISTOL-DALSTEIN (Geriatrician, Montpellier), Jean-Yves NIEMIER (Geriatrician, Nancy), Frédérique Retornaz (Geriatrician, Marseille), Véronique SERVENT (Medical Oncologist, Lille).

## Abbreviations

ADL: Activities of daily living
BMI: Body mass index
CGA: Comprehensive geriatric assessment
EORTC: The European Organization for Research and Treatment of Cancer
GDS: Geriatric depression scale
HR: Hazard ratio
HRQoL: Health related quality of life
IADL: Instrumental activities of daily living
IDMC: Independent data monitoring committee
ITT: Intent-to-treat
mITT: Modifiedodified intent-to-treat
MMSE: Mini mental state examination
MNA: Mini nutritional assessment
OS: Overall survival
PFS: Progression-free survival
RCTs: Randomized control trials
SPPB: Short performance physical battery
TUDD: Time until definitive deterioration
TUG: Timed up and go
